# Electrospun Poly(ε-caprolactone) Nanofibrous Mesh for Imiquimod Delivery in Melanoma Therapy

**DOI:** 10.3390/polym10030231

**Published:** 2018-02-26

**Authors:** Wei-Chih Lin, I-Ting Yeh, Eri Niyama, Wan-Rou Huang, Mitsuhiro Ebara, Chieh-Shan Wu

**Affiliations:** 1 Department of Mechanical and Electro-Mechanical Engineering, National Sun Yat-sen University, Kaohsiung 80424, Taiwan; m053020013@student.nsysu.edu.tw (I-T.Y.); hellorou777@gmail.com (W.-R.H.); 2 International Center for Materials Nanoarchitectonics (WPI-MANA), National Institute for Materials Science (NIMS), 1-1 Namiki, Tsukuba, Ibaraki 305-0044, Japan; niyama.eri@nims.go.jp (E.N.); ebara.mitsuhiro@nims.go.jp (M.E.); 3 Department of Dermatology, Kaohsiung Veterans General Hospital, Kaohsiung 81362, Taiwan; dermawu@vghks.gov.tw; 4 Department of Dermatology, Faculty of Medicine, College of Medicine, Kaohsiung Medical University, Kaohsiung 80708, Taiwan

**Keywords:** electrospinning, poly(ε-caprolactone), imiquimod, melanoma

## Abstract

Drug delivery systems (DDS) are commonly employed to administer drug-loaded composites to their therapeutic targets both in vitro and in vivo. Thus, we herein report the study of imiquimod-poly(ε-caprolactone) (IMQ-PCL) nanofibrous meshes for application in melanoma therapy. The preparation route employed was based on the electrospinning technique, with the melanoma cells being cultured on electrospun nanofibrous meshes to study their biocompatibility. All parameters employed, including the flow rate and polymer solution concentration, were examined to gain an improved understanding of the factors influencing the diameter and morphology of the electrospun fibre. The optimised parameters were employed to produce 12 IMQ-PCL nanofibrous meshes with diameters ranging from 100 to 900 nm to the melanoma cell viability. The relationship between the fibrous diameter and the imiquimod release profile was also determined using UV-Vis spectroscopy. In addition, similar results were obtained for the simulated imiquimod release profile obtained by COMSOL Multiphysics^®^. The IMQ-PCL nanofibrous meshes were found to decrease cell viability by ≥50%, with the number of cells dropping by ~10% over 48 h. As the cell viability was affected by the release of imiquimod, we believe that IMQ-PCL nanofibrous meshes are a promising drug delivery system for application in melanoma therapy.

## 1. Introduction

In recent decades, electrospinning (ES) has been employed extensively in the fabrication of ultra-fine nanofibrous matrices [1,2,3,4,5]. Indeed, the recent growing interest in ES can be attributed partly to the potential of nano-manufacturing for application in tissue engineering due to its ability to produce nano- to microscale meshes. As such, numerous characteristics of the ES technique, including the spinning mechanism, have been investigated. ES is a particularly attractive process due to its ability to produce nanofibrous matrices with diameters ranging from 40 to 4000 nm in a facile and straightforward manner, and also due to the improvements over crystalline or other similar systems [6]. The resulting electrospun nanofibrous matrices exhibiting a range of fibre diameters, surface morphologies, and mechanical strengths can then be employed in various diverse applications. In particular, their application in tissue engineering and regenerative medicine is of particular interest, as they mimic the nanoscale properties of the native extracellular matrix structure [7]. 

Due to these interesting properties, the fibre matrix can be regarded as a potential drug carrier. In this context, drug delivery systems (DDS) have attracted widespread interest in recent years [8,9,10,11,12]. More specifically, drug delivery systems consist of a formulation or device that enables the introduction of a therapeutic agent within the body, thereby enhancing its efficacy and safety by controlling the rate, time, and site of release. This system is essentially aimed at delivering and retaining sufficient quantities of an active drug molecule within an adequate period of time, and it is also expected to prevent the degradation of non-released drugs within the body [13,14,15]. As a result, the adverse effects associated with undesired fluctuations in drug concentrations and the ineffectiveness of degraded drug molecules can be alleviated.

In general, drug carriers are commonly employed in cancer therapy and tissue regeneration [1,16,17,18] to address issues relating to drug permeability. For example, an ideal dressing for use in tissue regeneration would mimic the functions of native skin. More specifically, this dressing should protect the injury from the loss of fluid and proteins, enable the removal of exudates, inhibit exogenous microorganism invasion, and improve the aesthetic appearance of the wound site. In other words, the biocompatibility and healing ability of microenvironments are crucial properties that should be considered during the development of ideal dressings [19,20,21]. Thus, a polymeric drug carrier based on polyester was previously reported to satisfy these conditions [17,19,20,21,22,23]. In this context, polycaprolactone (PCL), which has been approved by the Food and Drug Administration (FDA) as a biocompatible material, has been utilised in the preparation of slowly degrading wound dressings. Moreover, PCL is not only a skin-friendly material, but also allows the facile immobilisation of pharmaceutical drugs [5,20,24,25,26] via chemical modification. The PCL nanofibres and nanofibrous meshes are considered the promising candidate for tissue engineering and biomedical applications, due to several advantages such as a great surface-to-volume ratio [27,28]. A research team demonstrated that the PCL fibrous meshes possessed the higher drug permeability than the PLA matrix [29,30]. As such, synthesised PCL meshes can be considered ideal carriers in the context of the drug delivery system.

For the treatment of melanomas, although topical treatments [31] are commonly employed as non-surgical treatment methods, other options such as radiotherapy [32], photodynamic therapy [33], and cryotherapy are advantageous. One example of a topical treatment is imiquimod, which induces the production of a range of cytokines and chemokines (e.g., interferon-α) through activation of the Toll-like Receptor 7 pathway [34,35,36]. To date, various dosages and treatment periods have been shown to be effective [37,38,39]. In particular, Geisse et al. [38] demonstrated clearance rates of 75% and 73% over a six week period based on five applications per week and seven applications per week, respectively. 

Thus, we herein report determination of the optimal ES parameters for the preparation of nanofibres and subsequent investigation of the drug release profiles achieved using different fibre diameters. The working mechanism of the ES technique is based on the electrostatic driving forces that create an electrically charged polymer fluid jet between the polymeric solution and the collection plate by applying a high voltage bias to form micro/nano-scaled polymeric structures. In this case, the drug-loaded fibres will be electrospun using hexafluoro-2-isopropanol as the solvent. In addition, cell viability tests will be conducted using the Alamar Blue assay, the fibre characteristics will be examined by scanning electron microscopy (SEM), while the in vitro drug release behaviour of the fibres will be determined by UV-Vis spectroscopy. 

## 2. Materials and Methods 

PCL (*M_w_* = 45 kDa) was employed as the base material to manufacture the micro/nanoscale fibrous meshes according to a previously reported procedure [40]. To prepare the polymeric solution for the ES process, PCL and imiquimod (Sigma-Aldrich, Kaohsiung, Taiwan) were dissolved in 1,1,1,3,3,3-hexafluoro-2-propanol (HFIP, Sigma-Aldrich, Kaohsiung, Taiwan) and the resulting solution was poured into a transparent glass vial and stirred at 25 °C overnight. The various parameters employed during the preparation of the PCL solution are presented in Table 1. Phosphate buffered saline pH 7.4 (PBS, Sigma-Aldrich, Kaohsiung, Taiwan) was used to mimic the conditions within the human body during the drug release test.

### 2.1. Prepration of the PCL-Imiquimod Fibrous Meshes

#### 2.1.1. Preparation of the PCL-Imiquimod Solution

Three phases are known to exist where the stability of the mesh-incorporated imiquimod must be preserved, namely mesh fabrication, mesh storage, and mesh degradation [9], and so these phases must be considered during preparation of the fibrous meshes. Thus, the solutions required for the ES process were prepared by dissolving the required quantities of imiquimod and polymer in HFIP. More specifically, imiquimod (171 mg and 342 mg) which was calculated via the ratio of imiquimod and PCL, as shown in Equation (1) were added to HFIP (5 mL) in a clean glass vial and stirred at 25 °C for 1 h in an ultrasonic bath until a transparent solution was obtained. The evaluation of imiquimod after immersion in the electrospinning solvent (HFIP) were presented in our previous study [40,41]. After this time, PCL powder (3.42 g) was slowly added for preparing polymeric solution concentration of 30 wt %. Other concentration of polymeric solutions was calculated and prepared via the Equation (2). The resulting solution was stirred overnight at RT in the glass vial, which was sealed with a rubber cap to prevent solvent evaporation.

(1) $$\mathrm{Imiquimod}\;\mathrm{concentration}\;\left(\%\right)=\frac{W_{\mathrm{imiquimod}}}{W_{\mathrm{PCL}}}\times 100\%$$

(2) $$\mathrm{Solution}\;\mathrm{concentration}\;\left(\mathrm{wt}\;\%\right)=\frac{W_{\mathrm{PCL}}}{W_{\mathrm{PCL}}+W_{\mathrm{HFIP}}}\times 100\%.$$

#### 2.1.2. Preparation of the PCL Nanofibrous Meshes

**Figure 1:**
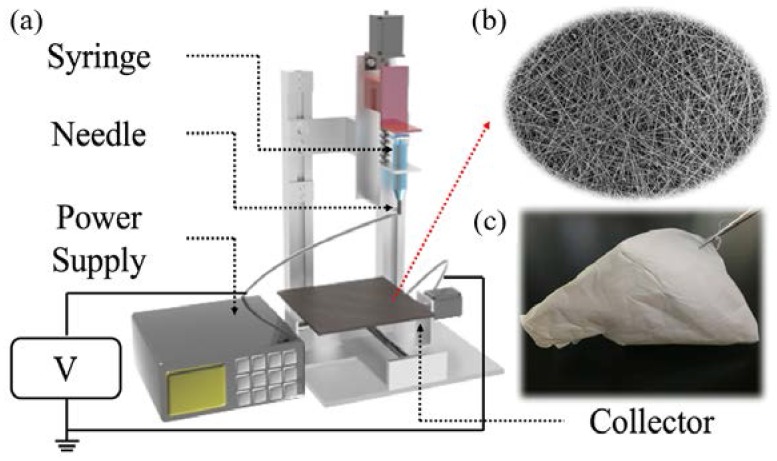
Preparation of the IMQ-PCL nanofibrous mesh: (**a**) Schematic representation of the ES process; (**b**) SEM image of the IMQ-PCL nanofibrous meshes; (**c**) The IMQ-PCL nanofibrous mesh obtained from the copper collector.

For the purpose of the ES process, the prepared imiquimod-polymeric solution was taken up into a 5-mL plastic syringe equipped with a stainless-steel capillary (18 and 23 G). A DC power supply (Nanon-01A, MECC Co., Ltd., Fukuoka, Japan) was utilised to provide a high voltage between the capillary and the copper collection plate. For all experiments, the applied voltage was set at 10–20 kV, the capillary-to-collector distance was 13–19 cm, and the flow rate was 0.1–0.9 mL/h. All processes were conducted at 26 ± 3 °C and the relative humidity (RH) was control at 50%. The obtained samples were then wrapped in aluminium foil and stored in a desiccator for ≥12 h prior to analysis. A schematic representation of the ES process is depicted in Figure 1.

**Figure 2:**
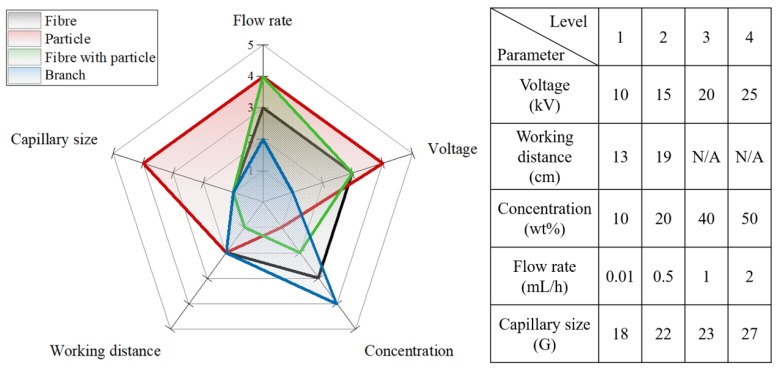
Optimisation of the parameters employed during preparation of the IMQ-PCL nanofibrous meshes and their effect on the morphology of the resulting IMQ-PCL nanofibrous mesh.

During the ES process, several key parameters exist, including the flow rate, the polymer solution concentration, the voltage, the working distance, and the capillary size. Thus, >130 samples were prepared during optimisation of these parameters for drug release, as indicated in Figure 2. Each parameter was divided into 4 levels which can indicate the effect on the morphology of the resulting IMQ-PCL nanofibrous mesh. For example, the level 1 in the voltage category represents a low biased voltage, which is the 10 kV applied during the ES process. As can be seen in the red region from Figure 2, applying the high level of the flow rate, voltage and capillary size are necessary factors for fabricating ball geometry of PCL particles. Similarly, the black, green and blue regions represent the electrospun PCL fibre, fibre with particle and branch, respectively. The SEM images in Section 3.1 show the different geometries of PCL structures which were fabricated by applying the parameters. In addition, the result, as shown in Section 3.2, indicated that the nanofibrous mesh prepared using four distinct types of cells yielded a suitable biomedical mesh for tissue engineering. As such, these ES parameters were employed to prepare the desired IMQ-PCL nanofibrous meshes nanofibre for the treatment of melanoma cells.

#### 2.1.3. Preliminary Study into Biocompatibility of the PCL Nanofibrous Meshes

Fluorescein diacetate (FDA, Sigma-Aldrich, Kaohsiung, Taiwan) staining [42] would infiltrate the cell membrane and remains colourless. When the acetate moieties in FDA are laminated by esterizes, the non-fluorescent FDA was converted to fluorescent state [43]. Therefore, the viability of cells can be readily detected due to the visualization of viable cells with the intracellular FDA which is exposed to ultraviolet light (366 nm) [44]. In this study, the proliferation of four kinds of cells, including NIH-3T3 mouse embryo fibroblast cell line (NIH-3T3, BCRC, Hsinchu, Taiwan), ER positive human breast cancer cells (MCF-7 cells, BCRC, Hsinchu, Taiwan), Human malignant melanoma cells (BCRC, Hsinchu, Taiwan), and human oral squamous cell carcinoma (SAS cell line, BCRC, Hsinchu, Taiwan), were observed by FDA staining approach. Briefly, the PCL nanofibrous meshes were firstly cut into circle shape with 15.6 mm diameter, and then were placed in a 24-well culture plate. Each kind of cell (1 × 10^4^ per mesh) was seeded on the PCL nanofibrous meshes and incubated at 37 °C and 5% CO_2_. At specified time intervals (i.e., Day 1, 3, and 5), the PBS was used to wash the samples and the same process was repeated three times. The0.5 mL of 4% glutardialdehyde (Sigma-Aldrich, Kaohsiung, Taiwan) was employed to fix the cells for 10 min after the medium was withdrawn. Then, samples were washed three times by PBS, and 0.5 mL of the freshly diluted solution of FDA (5 mg/mL) in phosphate buffered saline (PBS) was added and placed in incubator for 10 min. A photograph of each cell was scanned and analysed using microscope with NIS-Elements AR software (Eclipse Ni-U, Nikon, Tokyo, Japan). 

### 2.2. Characteristics of the Imiquimod-Nanofibrous Meshes

Scanning electron microscopy (SEM, SU-8000, Hitachi, Tokyo, Japan) was employed to observe the morphology of the prepared fibres. The various samples were separated from the aluminium foil and were cut into squares measuring 1 cm × 1 cm. To increase their electronic conductivity, the samples were fixed on the SEM stub using carbon tape and were sputter-coated with titanium prior to imaging. The diameters of the obtained fibres (over 200 randomly selected segments) were determined using Image-J software [45] and the average values calculated. 

### 2.3. Evalulation of the In Vitro Imiquimod Release Profiles

As the duration of imiquimod release has a significant effect on the proliferation of cancer cells, the release mechanism of the active component from the IMQ-PCL nanofibrous meshes prepared herein was examined. 

Initially, the various samples were cut into 1.5 cm^2^ segments to eliminate variation in drug release caused by non-uniform fibre distribution in the same mesh samples due to the random manner of the ES process. Each sample (prepared in triplicate) was weighed (2–7 mg) using an electro-balance (BSA224S-CW, Sartorius, Gottingen, Germany) and then placed into a pre-washed 24-well culture plate. Pre-warmed PBS was added to each well and the plate was placed in an incubator at 37 °C to mimic the conditions seen in the human body. At specified time intervals, an aliquot of solution (1 mL) was removed from each well and replaced with fresh, pre-warmed PBS to maintain the same volume throughout the release test. The various samples were then analysed by UV-Vis spectroscopy (V-770 spectrophotometer, Jasco, Tokyo, Japan) at λ = 244 nm to determine the imiquimod concentration. All UV-Vis data were converted into the corresponding imiquimod concentrations using the appropriate calibration curve showed in Equation (3), and the cumulative amounts of released drug were calculated as a function of the incubation time, as outlined in Equation (4).

(3) y=0.5397x+0.045

where *y* is defined as the absorbance value and the *x* represents the imiquimod concentration in PBS.

(4) $$M_{t}=C_{t0}V_{\mathrm{PBS}}+\sum^{\text{​}}C_{t}V_{\mathrm{PBS}}.$$

where *C_t_*_0_ is defined as the concentration of drug released in the PBS solution at time = 0, *C_t_* is the concentration of drug released in the PBS solution at time (*t*), and *V*_PBS_ is the volume of the PBS solution employed.

### 2.4. Cell Cultures and In Vitro Cytotoxicity Assays

To examine the effect of imiquimod on cell proliferation, the Alamar Blue assay was utilised for the in vitro cytotoxicity assay. Briefly, the samples were firstly sterilized for 24 h via the UV light. The melanoma cells derived from ATCC were then cultured in Dulbecco’s modified Eagle’s medium supplemented with 20 vol % FBS and 1 vol % antibiotic-antimycotic. The melanoma cells (1 × 10^4^ cells per well) were seeded in 24-well tissue culture plates (Falcon, Bedford, MA, USA) and incubated at 37 °C in a humidified atmosphere containing 5% CO_2_. After 24 h, the pre-sterilised samples were utilised to cover the melanoma cells for the purpose of mimicking a mesh. Subsequently, the Alamar blue solution was added to each well, and an enzyme-linked immunosorbent assay (ELISA) reader was utilised to measure the colorimetric changes at an absorbance of 530/590 nm after 90 min.

### 2.5. Simulation of the Imiquimod Release Profile

**Figure 3:**
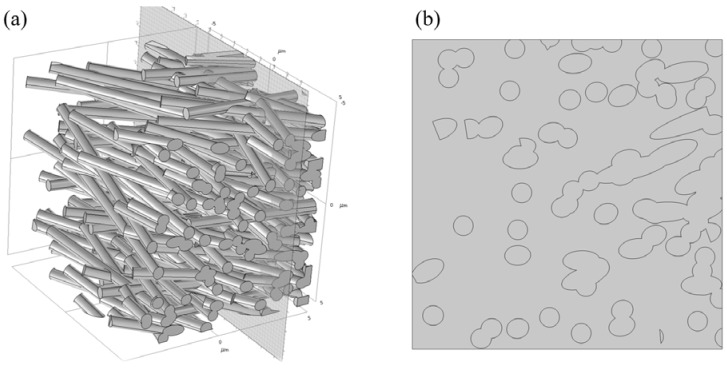
The designed model for simulation of imiquimod release profile: (**a**) the 225 cylindrical fibres randomly generated in a cubic; (**b**) the setting cross section of the designed model.

To analyse the release of imiquimod from the PCL nanofibrous meshes, simulations were carried out in a 125 µm^3^ cubic model using COMSOL Multiphysics^®^ (COMSOL Server™ Version 5.3a, PITOTECH, Taipei, Taiwan) [46]. The dimension of the element results from observations of the typical thicknesses of the PCL nanofibrous meshes was set to L = 100 µm. To mimic the real nanofibrous mesh, 225 cylindrical fibres were randomly placed in the cubic element, as shown in Figure 3. The average diameters of the random fibres are given Table 2, and these values were comparable to the experimental values obtained, as determined by comparing the simulated average specific diameters with those of the experimentally-obtained polymers.

The transient sorption process applied in the numerical model is a Langmuir bimolecular process between drug molecule *C_A_* and an unoccupied site (*C_Bmax_* − *C_B_*), where *C_Bmax_* is the maximum concentration of adsorbed drug. The following necessary assumptions have been applied in the model: (1) the mechanism of diffusion followed Fick’s law, as shown in Equation (3); (2) all diameters of the fibres in the cubic model were equivalent; (3) each empty adsorption site was equivalent; (4) a homogenous distribution was set for imiquimod at the beginning of the release process; and (5) degradation of the PCL nanofibres did not occur during the release period. In addition, the change in drug concentration on the fibre surface can be represented by adsorption and desorption terms with rate constants of *k_ads_* and *k_des_*, respectively:

(5) $$\frac{\partial C_{B}}{\partial t}=k_{ads}\times C_{A}\times \left(C_{Bmax}-C_{B}\right)-k_{des}\times C_{A}$$

where *C_B_* is the drug concentration expressed in relation to the weight of the polymer material (kg/kg), *C_A_* is the drug concentration in the fluid (kg/m^3^), the *C_Bmax_* obtained from the experimental results was set to 1.37006 × 10^−1^, and *k_ads_*, *k_des_*, and *k_c_* were 10^−6^, 10^−5^, and 10^−9^ [47], respectively.

## 3. Results

Recently, the development of electrospun nanofibrous mesh for use as a drug delivery system (DDS) in medical applications has received growing attention. Based on previous work, it was apparent that the polymeric solution is of particular importance as it allows preparation of the drug-loading fibrous mesh via ES prior to loading of the desired drug molecule. We therefore selected this approach for the purpose of our study. Following their successful preparation, the morphology, rate of drug release, and cell viability were characterised. 

### 3.1. Characterisation of the Fabricated IMQ-PCL Nanofibrous Mesh

The orthogonal experimental method was used to investigate the effect of the system parameters on the diameters and morphologies of the electrospun meshes, and the results are presented in Table 1.

**Figure 4:**
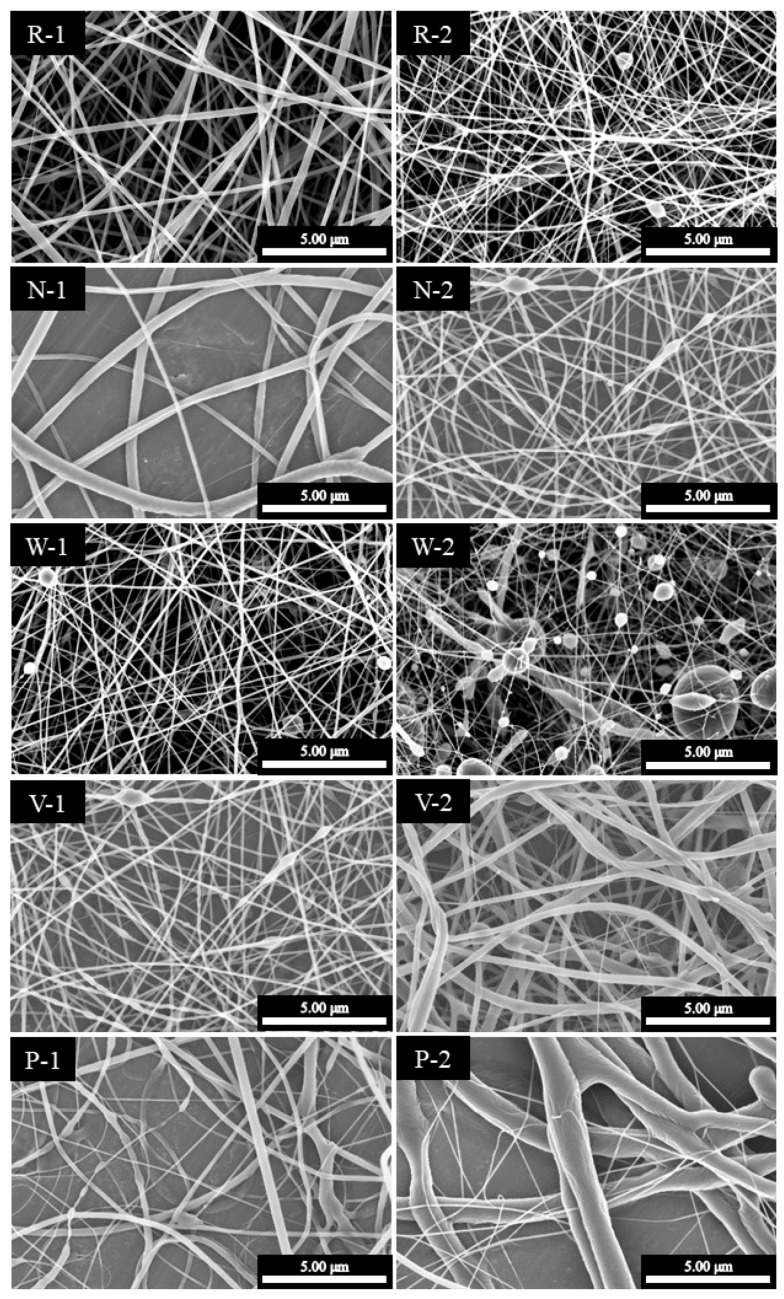
SEM images of the IMQ-PCL nanofibrous meshes. The parameters employed to prepare each sample (R-1 to P-2) are outlined in Table 1.

The effect of the polymeric solution concentration was initially investigated due to its role in determining the mesh structure. Based on the obtained results, it was apparent that a low concentration resulted in a smooth polymeric flow at the end point of the capillary, thereby leading to a highly viscous ES state and the formation of fibrous structures. In contrast, a particulate structure was obtained when the concentration was decreased to a critical value. Furthermore, the flow rate also affected the formation of the particulate structure at equivalent polymeric concentrations, as indicated in Figure 4 (W-2).

**Figure 5:**
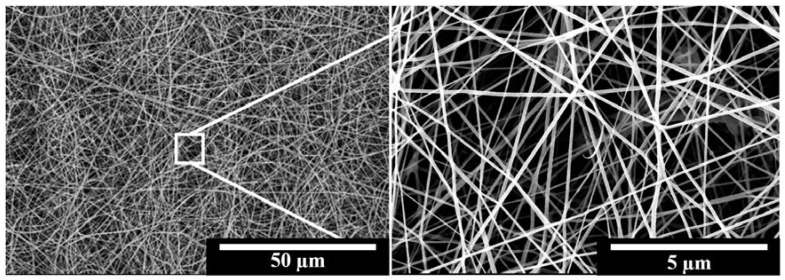
SEM images of the IMQ-PCL nanofibrous mesh prepared using the optimised parameters (i.e., 30 wt % polymer solutions, 20 kV applied voltage, 18 G capillary).

It was also found that the diameters of the majority of fibres ranged from 200 to 1200 nm. The relationship between the parameters employed and the mesh structures are outlined in Figure 2, as described previously. Indeed, the desired fibrous structure was easily produced upon increasing either the size of the capillary or the applied voltage. The concentrations of the polymeric solution also play a key factor in the fabricated geometries of the PCL structures, such as the particle, fine and branch fibres. Finally, a polymeric solution concentration of 30 wt % with 20 kV applied voltage and 18 G capillary produced the optimal PCL nanofibrous meshes, as shown in the SEM images provided in Figure 5. 

### 3.2. Preliminary Study into Biocompatibility of the PCL Nanofibrous Meshes

**Figure 6:**
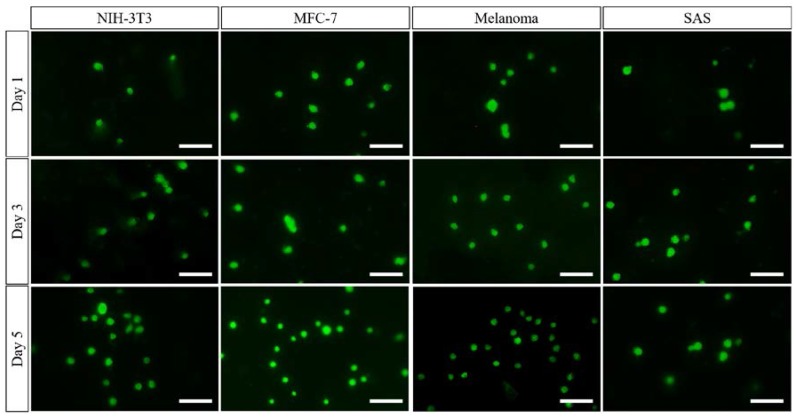
Fluorescent images of the four kinds of cells that were cultured on the optimised nanofibrous meshes after five days. The images showed the proliferation and spreading of four kinds of cells (i.e., MCF-7 cells, NIH-3T3 fibroblast cells, SAS cells, and melanoma cells) which supported the biocompatibility of PCL nanofibrous meshes. The scale bar is 50 μm.

During preparation of the PCL nanofibrous meshes, it was apparent that the key parameters were the polymeric concentration, the PCL solution flow rate, and the applied voltage. As shown in Figure 5, the PCL nanofibrous mesh exhibited an ultrafine morphology using the optimal parameters discussed in Section 3.1. In this case, the fibres were separated on the copper collector, with no particulate formation being observed. As the purpose of this mesh is for biological application within the human body, a low toxicity is essential. Cell viability tests were therefore carried out, and it was confirmed that the PCL nanofibrous mesh exhibited excellent biocompatibility. More specifically, as shown in Figure 6, four kinds of cell (i.e., MCF-7 cells, NIH-3T3 fibroblast cells, SAS cells, and melanoma cells) spread and exhibited continuous proliferation over five days. The increasing cell numbers for each sample suggest that the PCL nanofibrous meshes possess excellent biocompatibility.

### 3.3. Release Profiles of the Loaded Imiquimod

**Figure 7:**
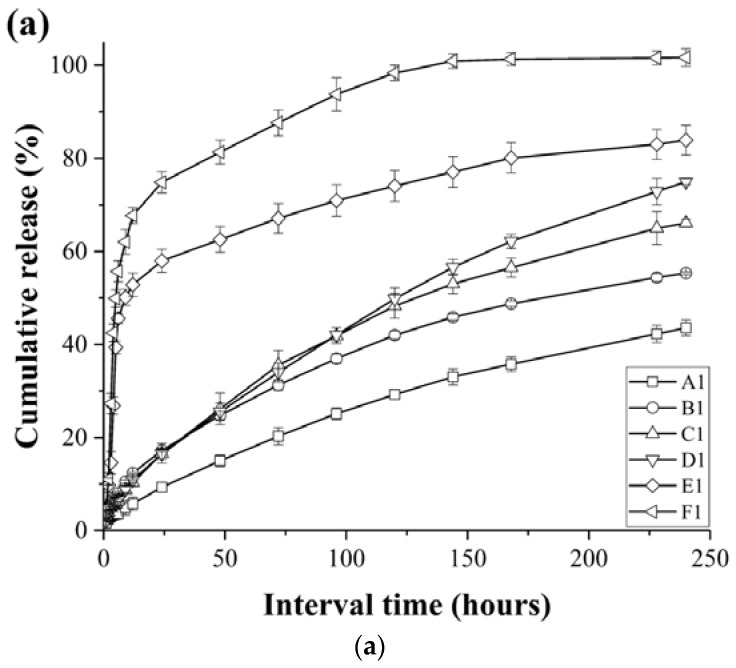

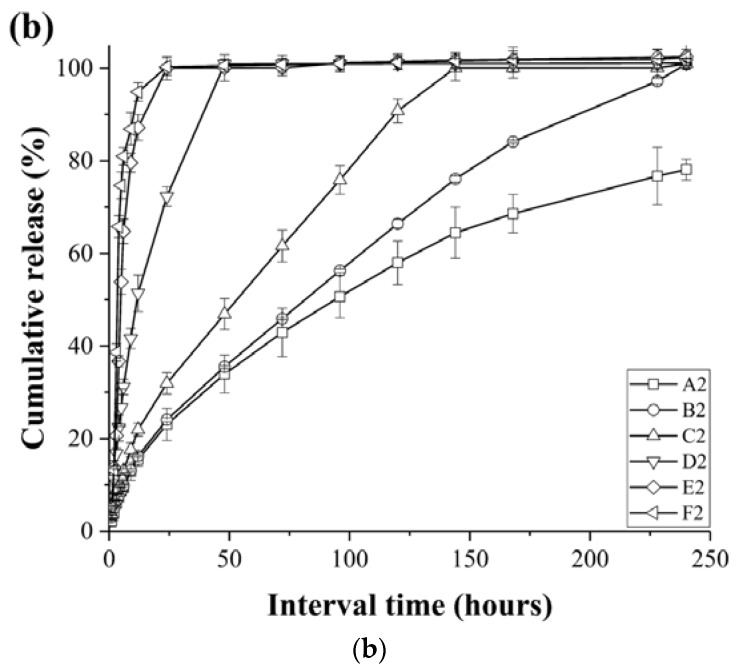
Imiquimod release profiles (**a**) 5 *w*/*w* % and (**b**) 10 *w*/*w* % for the various PCL nanofibrous mesh samples (A1 to F2) described in Table 2.

In the context of drug carriers, the drug-release conditions are of particular importance, and so they have received significant attention in recent decades. Thus, we herein carried out rigorous testing of the drug release behaviour of 12 PCL nanofibrous meshes containing two different imiquimod loadings (5 and 10 wt %) under in vitro release conditions over 244 h (~10 days), as shown in Figure 7a,b. The detailed characteristics of the mesh samples are presented in Table 2. As indicated, after 244 h, samples A1, B1, C1, D1, E1 and A2 gave cumulative drug release percentages of 43.5, 55.3, 66.1, 70.7, 83.9 and 74.9%, respectively. Interestingly, in the initial 24 h of drug release, each sample exhibited a slight “burst release.” In addition, as the concentration of imiquimod decreased in the PCL nanofibrous meshes, drug release slowed, and reached a plataux for a number of samples between 150 and 240 h. Furthermore, Figure 7a,b indicate that in the fibres with larger diameters, the IMQ-PCL nanofibrous meshes exhibited faster drug release, with superior results being observed for the mesh samples containing 10 wt % imiquimod. The samples of F1, B2, C2, D2, E2 and F2 performed 100% cumulative release as shown in Figure 7a,b.

We expect that these phenomena may be attributed to both the surface area to volume ratio (SA:V) and the diffusion mechanism of the mesh samples. Based on the results discussed in Section 3.1 and Section 3.2, an increase in the flow rate employed during preparation of the IMQ-PCL nanofibrous meshes led to larger diameters. In addition, under a constant volume, smaller diameters resulted in reduced surface areas due to the layers of the fibre structures overlapping. Thus, the IMQ-PCL nanofibrous meshes with large diameters exhibited high surface area to volume ratios, and so imiquimod exchange occurred rapidly and its diffusion was enhanced. In contrast, the IMQ-PCL nanofibrous meshes with small diameters exhibited smaller surface area to volume ratios, and so imiquimod exchange was less efficient, thereby leading to a decrease in the rate of imiquimod diffusion due to the larger distances involved in its transport.

Furthermore, the presence of high imiquimod concentrations in the PCL nanofibrous meshes resulted in enhanced release rates. This can be accounted for by the diffusion element of drug release involving the movement of molecules or atoms from a region of high concentration to a region of low concentration. Thus, greater differences between the high concentration potential and the low concentration potential resulted in quicker release rates, as indicated in Figure 7a,b. 

### 3.4. Effect of the IMQ-PCL Nanofibrous Mesh on the Proliferation of Melanoma Cells

**Figure 8:**
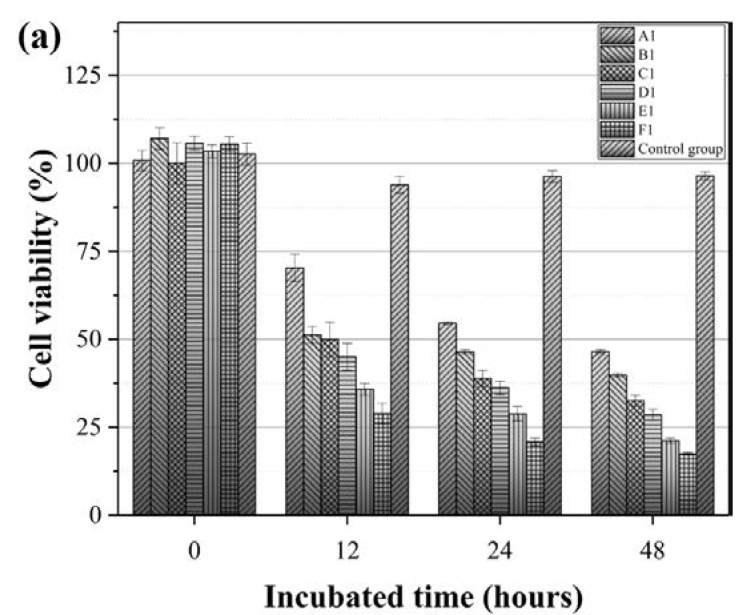

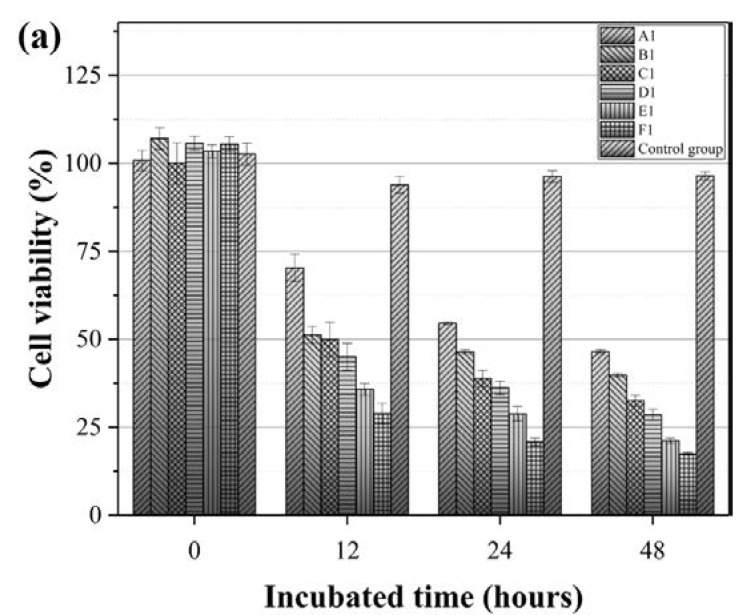
Melanoma cell viability following culture on the (**a**) 5 *w*/*w* % and (**b**) 10 *w*/*w* % IMQ-PCL nanofibrous meshes over 48 h (*n* = 3).

The effect of drug release rates on cell proliferation is also a key point when considering the use of nanofibrous meshes as potential drug carrier systems. As such, melanoma cells were employed to investigate the effect of the imiquimod release rates described in Section 3.3 on cell proliferation (see Table 2 for sample details). Thus, Figure 8 outlines the resulting melanoma cell viabilities after culturing on the IMQ-PCL nanofibrous meshes for 6, 12, 24, and 48 h. All samples (Figure 8 A1–F1 and A2–F2) presented a degree of inhibition in the melanoma cell viability test. More specifically, in the initial 6 h, cell proliferation continued; however, beyond this point, the cell viability began to decline in all samples due to the release of imiquimod from the mesh. In the case of the samples containing 5 wt % imiquimod, slightly higher cell viabilities were observed due to the larger diameters of the mesh and the lower drug loading, with cell numbers dropping by ~50–70% within two days. Interestingly, although a similar trend was observed for the mesh samples containing 10 wt % imiquimod, a more significant reduction in cell viability was observed. These results suggest that the proliferation of melanoma cells is influenced by the differing drug release rates, which were previously discussed in Section 3.3. More specifically, higher drug release rates led to significant reductions in melanoma cell proliferation, thereby suggesting that the IMQ-PCL nanofibrous mesh is a promising drug carrier system for application in the treatment of melanomas.

### 3.5. Simulation of the Imiquimod Release Profile

**Figure 9:**
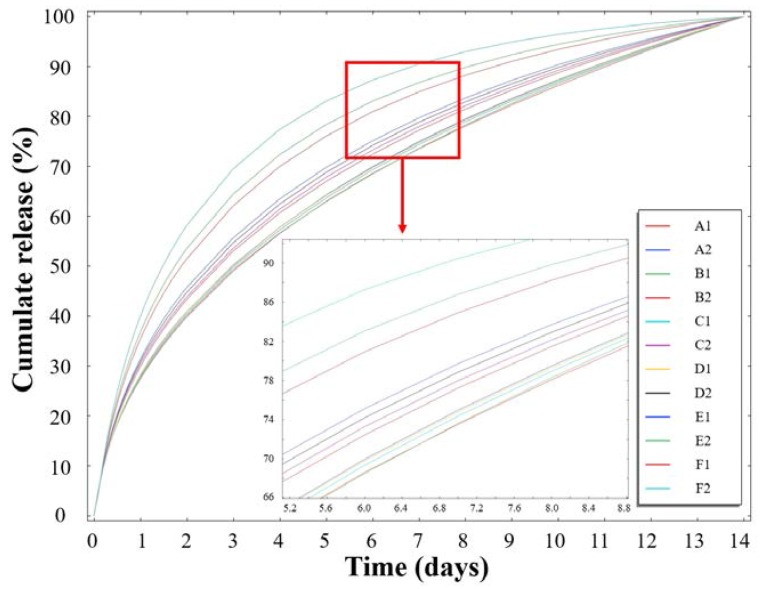
Simulation of imiquimod release under different drug loadings and mesh diameters (see Table 2 for sample identification).

To confirm the optimal conditions required for imiquimod release, the experimental data were imported to COMSOL Multiphysics^®^ and COMSOL Server™ Version 5.3a and simulated under the designed model using the parameters outlined in Table 2. As indicated in Figure 9, all samples exhibited a degree of burst release in the initial 48 h. In addition, drug release was accelerated upon increasing the fibre diameter and the imiquimod loading. These phenomena could be contributed to the spontaneous diffusion of the imiquimod present in the nanofibrous meshes. However, a number of differences existed between the simulated results and the experimental results. For example, in the case of samples A2 and C2 outlined in Figure 7, imiquimod release was complete after 48 and 144 h, respectively, with this difference being attributed to polymer degradation and the distribution of imiquimod in the nanofibrous mesh. This differed from the ideal imiquimod release observed in the simulation. More specifically, polymer degradation accelerated imiquimod release, and the imiquimod molecules close to the nanofibre edges were easily released. However, in general, the simulation results supported the experimental results described in Section 3.3.

## 4. Conclusions

We herein described the preparation of imiquimod-poly(ε-caprolactone) (PCL) nanofibrous meshes using the electrospinning approach for application as a drug release system in melanoma therapy. Optimisation of the electrospinning parameters, including the flow rate and PCL solution concentration, was carried out to prepare nanofibrous meshes with diameters ranging from 140 to 1200 nm. This diameter was controlled using the electrospinning flow rate, and the drug release rate was influenced by the mesh diameter. In addition, two different imiquimod concentrations (i.e., 5 and 10 wt %) were examined, with the mesh samples containing higher drug loadings giving greater reductions in cell viability. Following examination of the imiquimod release profiles of a number of samples using UV-Vis spectrometry over ~10 days, it was apparent that the samples containing smaller diameters and lower imiquimod loadings exhibited more stable imiquimod release rates and quantities. In addition, simulation of the imiquimod release profile provided similar results to those obtained experimentally under comparable conditions. Furthermore, following culture of the melanoma cells on the PCL fibrous meshes and the IMQ-PCL nanofibrous meshes over 48 h, essentially no changes in cell viability were observed for the pure PCL nanofibrous mesh. However, the melanoma cells cultured on imiquimod-containing fibres exhibited a ≥50% decrease in cell viability and a 10% decrease in cell numbers between 6 and 48 h. Thus, the obtained experimental results indicate that the electrospun PCL nanofibres prepared herein are a suitable candidate for application as an imiquimod carrier in the treatment of melanomas.

## Figures and Tables

**Table 1:** Average diameters of the prepared imiquimod-poly(ε-caprolactone) nanofibrous meshes and the electrospinning parameters employed herein.

Recipe	Flow Rate (mL/h)	Capillary Size (G)	Voltage (kV)	Polymeric Solution Concentration (wt %)	Working Distance (cm)	Average Diameter (nm)
R-1	0.01	23	10	30	13	104 ± 40
R-2	0.25	23	10	30	13	172 ± 59
N-1	0.01	18	10	30	13	321 ± 172
N-2	0.01	23	10	30	13	119 ± 35
W-1	0.5	23	10	30	13	101 ± 37
W-2	0.5	23	10	30	19	90 ± 34
V-1	0.01	23	10	30	13	126 ± 32
V-2	0.01	23	20	30	13	249 ± 172
P-1	0.05	23	10	20	13	194 ± 21
P-2	0.05	23	10	30	13	1341 ± 504

**Table 2:** Parameters of the prepared imiquimod-poly(ε-caprolactone) nanofibrous meshes employed in determination of the drug release profile.

Samples	Flow Rate (mL/h)	Imiquimod Loading (*w*/*w* %)	Polymeric Solution Concentration (wt %)	Diameter (nm)
A1	0.225	5	30	268 ± 21
A2		10	30	273 ± 21
B1	0.450	5	30	281 ± 16
B2		10	30	284 ± 19
C1	0.675	5	30	296 ± 42
C2		10	30	291 ± 27
D1	0.900	5	30	318 ± 61
D2		10	30	335 ± 17
E1	1.35	5	30	714 ± 29
E2		10	30	702 ± 33
F1	0.900	5	40	967 ± 48
F2		10	40	959 ± 25
